# Two cases with intra‐aortic balloon pumping use for severe septic cardiomyopathy

**DOI:** 10.1002/ams2.292

**Published:** 2017-07-13

**Authors:** Taro Hiromi, Chiaki Toida, Takashi Muguruma, Katsutaka Hashiba, Tomoki Doi, Kyota Nakamura, Naoto Morimura

**Affiliations:** ^1^ Department of Emergency Medicine Yokohama City University Graduate School of Medicine Minami‐ku Yokohama Japan; ^2^ Cardiovascular Medicine Yokohama City University Graduate School of Medicine Minami‐ku Yokohama Japan

**Keywords:** Intra‐aortic balloon pump, septic cardiomyopathy, septic shock

## Abstract

**Cases:**

Septic cardiomyopathy is defined as a reversible left ventricular systolic dysfunction. Patients with severe septic cardiomyopathy have a high mortality rate, even if they receive conventional therapy. For those patients, previous reports showed intra‐aortic balloon pump (IABP) efficacy. We report two rare cases with IABP introduction leading them to drastic improvement, and survival from severe septic cardiomyopathy. Case 1 is a 78‐year‐old woman diagnosed with renal calculus pyelonephritis, septic shock, and septic cardiomyopathy. Case 2 is a 62‐year‐old man diagnosed with pneumonia, septic shock, and septic cardiomyopathy.

**Outcome:**

In both cases, despite conventional therapy for cardiomyopathy, including high‐dose catecholamine therapy, shock was not reversed, and the IABP was inserted. Circulatory status was improved after the introduction of the IABP.

**Conclusion:**

Our findings suggest that an IABP can be useful for salvaging patients with septic cardiomyopathy who do not respond to conventional therapy.

## Background

Septic cardiomyopathy is defined as a reversible left ventricular systolic dysfunction that occurs in patients with sepsis. Most patients improve with conventional therapy after 7–10 days^1^. The rate of septic cardiomyopathy is 18–65% among patients with sepsis and the mortality rate is 36–55%.^2^

The strategy of treatment for septic cardiomyopathy is generally the same as that for cardiomyopathy without sepsis, such as by fluid replacement and administration of catecholamine. Additional therapy is controversial for patients who do not respond to this conventional therapy. Although the intra‐aortic balloon pump (IABP) has been used in some cases, its efficacy remains controversial,^3^ and the outcomes have not been sufficiently reported.

We report two cases with IABP introduction leading to drastic improvement, and survival from severe septic cardiomyopathy when conventional therapy was not effective.

This study was approved by the ethics committee of Yokohama City University (Yokohama, Japan). Written informed consent was obtained from the subjects and/or guardians for publication of this report.

## Cases

### Case 1

The patient was a 78‐year‐old woman with shock who was transferred to our hospital on the day after ureterolithotomy.

Glasgow Coma Scale was E1V1M4, indicating disturbance of consciousness. She had tachypnea with a respiration rate of 26 breaths/min. Oxygenation was impaired, with SpO_2_ of 89% on oxygen at 6 L/min and heart rate was 115 b.p.m. A diagnosis of septic shock was made. The patient underwent tracheal intubation for mechanical ventilation and noradrenaline was given.

The results of laboratory tests showed: increased leucocytes (27,840/μL) and troponin‐I (1.1 ng/mL); blood urea nitrogen, 36 mg/dL; creatinine, 3.7 mg/dL; prothrombin time – international normalized ratio, 1.5; activated partial thromboplastin time, 43.3 s. Blood gas analysis revealed: pH 7.14; PaCO_2_, 63.6 mmHg; ${\text{HCO}}_{3}^{-}$, 21.1 mmol/L; lactate, 3.9 mmol/L. Cardiomegaly and pulmonary edema were observed on the chest X‐ray. Abdominal computed tomography showed a right renal calculi and pyelonephritis. The electrocardiogram revealed ST segment depression in lead aVR and ST segment elevation in leads V3–6. Echocardiography showed that the left ventricular ejection fraction (LVEF) was 25% with diffuse LV systolic dysfunction. Coronary angiography did not identify any significant coronary artery stenosis.

**Figure 1:**
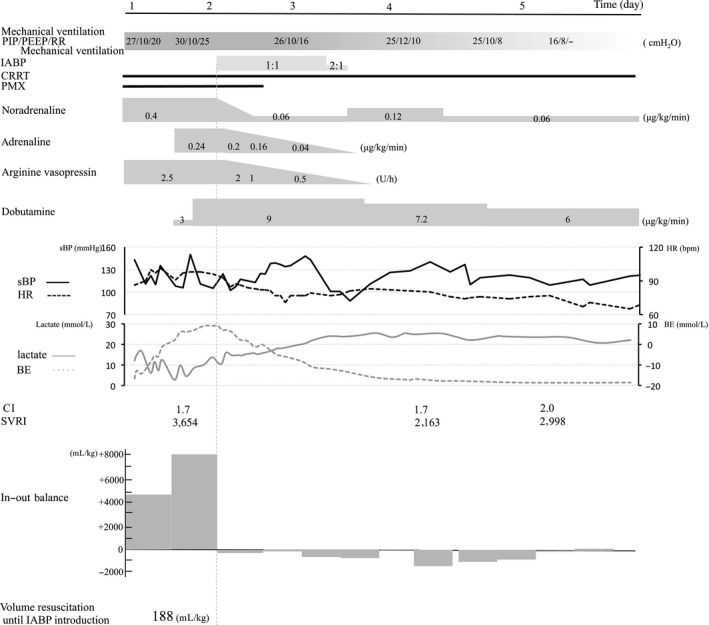
Clinical course from day 1 to day 5 in a 78‐year‐old woman with severe septic cardiomyopathy. Top panel, ventilator settings (mode, positive end‐expiratory pressure [PEEP], peak inspiratory pressure [PIP], and respiratory rate [RR]), duration of intra‐aortic balloon pump (IABP) use, continuous renal replacement therapy (CRRT), and direct hemoperfusion with a polymyxin B immobilized fiber column (PMX). Middle panel, catecholamine dose. Bottom panel, change in vital signs (systolic blood pressure [sBP] is shown by the solid black line; heart rate [HR] is shown by the broken black line), lactate level (solid gray line), base excess (broken gray line), cardiac index (CI), systemic circulation resistance index (SVRI), and fluid balance.

The patient was admitted to the intensive care unit (ICU) with renal calculus pyelonephritis, septic shock, and septic cardiomyopathy. Her course after admission is shown in Figure 1. As pressor agents, noradrenaline, arginine vasopressin, and dobutamine were administered. In addition, direct hemoperfusion with a polymyxin B immobilized fiber column was started and continuous renal replacement therapy was carried out for acute renal failure. Despite high‐dose catecholamine therapy, shock was not reversed. An IABP was inserted on day 2. Her vital signs became stable and the IABP was removed on day 3. On day 6, echocardiography (ECG) showed that her EF had improved to 60%. She was discharged from ICU on day 31 and was transferred to a rehabilitation facility on day 81.

### Case 2

The patient was a 62‐year‐old man who presented with high fever and impaired oxygenation. He was referred to our hospital for treatment of pneumonia.

The results of laboratory tests showed: decreased leucocytes, 2590/μL; elevated troponin‐I, 0.13 ng/mL; blood urea nitrogen, 35 mg/dL; creatinine, 1.7 mg/dL; prothrombin time – international normalized ratio, 1.3; activated partial thromboplastin time, 46.4 s. Blood gas analysis under 10 L/min oxygen inhalation revealed oxygenation failure (PaO_2_ 98.4 mmHg). The LVEF was 20% on ECG, and diffuse LV systolic dysfunction was observed. His chest X‐ray showed infiltration in the left lower lung field. Chest computed tomography revealed bilateral lower lobe pneumonia.

**Figure 2:**
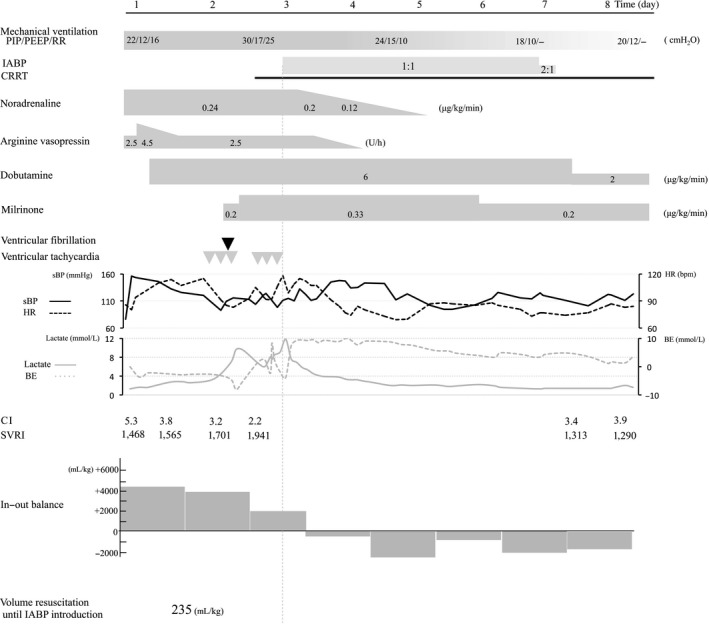
Clinical course from day 1 to 8 in a 62‐year‐old man with severe septic cardiomyopathy. Top panel, ventilator settings (mode, positive end‐expiratory pressure [PEEP], peak inspiratory pressure [PIP], and respiratory rate [RR]), duration of intra‐aortic balloon pump (IABP) use, continuous renal replacement therapy (CRRT), and direct hemoperfusion with a polymyxin B immobilized fiber column (PMX). Middle panel, catecholamine dose. Bottom panel, change in vital signs (systolic blood pressure [sBP] is shown by the solid black line; heart rate [HR] is shown by the broken black line), lactate level (solid gray line), base excess (BE; broken gray line), cardiac index (CI), systemic circulation resistance index (SVRI), and fluid balance. Gray and black triangles show the points when ventricular fibrillation and ventricular tachycardia occurred, respectively.

Tracheal intubation and mechanical ventilation were started for septic shock associated with pneumonia, as well as administration of noradrenaline, arginine vasopressin, and dobutamine. The patient was admitted to the ICU. The clinical course is shown in Figure 2. Treatment with a phosphodiesterase III inhibitor was started on day 2. Transient ventricular tachycardia and ventricular fibrillation occurred.

On day 3, continuous renal replacement therapy was initiated for acute renal failure. The catecholamine dose could not be reduced and life‐threatening arrhythmias occurred repeatedly. An IABP was inserted and catecholamine was subsequently tapered. The IABP was removed on day 7 when ECG showed that the patient's EF had improved to 35%. He was discharged from the ICU on day 27 and was referred to a rehabilitation facility on day 58.

## Discussion

The IABP was a highly effective device in our patients with severe septic cardiomyopathy.

Septic cardiomyopathy is defined as a reversible LV dysfunction in patients with sepsis. In these patients, myocardium is functionally and structurally injured by inflammatory cytokines and mitochondrial dysfunction. As a result, ECG shows LV dysfunction and dilatation without regional dysfunction. These findings are useful to describe the difference from ischemic cardiac failure.^1^, ^2^ In sepsis, both the peripheral oxygen requirement and cardiac output are generally increased. However, in patients with septic cardiomyopathy, the cardiac output becomes inadequate to supply the increased peripheral oxygen requirement and the patient's condition deteriorates. Finally, these patients have decompensated hear failure with severe LV dysfunction.

As with the treatment of septic shock, the conventional therapy for septic cardiomyopathy includes fluid replacement and administration of vasopressor agents. Recently, it has been suggested that phosphodiesterase III inhibitors may also be useful for septic cardiomyopathy.^1^, ^2^ Our patients did not respond well to conventional therapy. Previous reports have described that poor prognostic factors for septic cardiomyopathy include LVEF <40% and LV diastolic dysfunction (early diastolic velocity of mitral annulus [e’] <8.0 cm/s), and an inadequate response to vasopressor agents).^2^, ^8^ It was also reported that the mortality rate is higher than 60% if patients have both diastolic dysfunction and LV systolic dysfunction.^8^ Both of our patients had all of the poor prognostic factors (Case 1, LVEF 25% and e’ 5.34 cm/s; Case 2, EF 20% and e’ 2.87 s/cm); they may not have survived with conventional therapy alone.

Some case reports showed that veno‐arterial extracorporeal membrane oxygenation (ECMO) or IABP was useful as the last rescue therapy for unresponsive sepsis cardiomyopathy patients.^4^ Because the management of patients with ECMO is very complex, IABP might be useful as the bridging therapy until ECMO introduction. It has been reported that the IABP can improve the balance between myocardial oxygen supply and consumption, as well as improving the peripheral circulation.^5^, ^6^, ^7^ We suggest that the IABP is beneficial for septic cardiomyopathy patients who had decompensated heart failure with LV dysfunction because it reduces afterload, increases coronary blood flow, and also improves the coronary circulation.

However, it has been reported that complications can occur, such as thrombocytopenia and bleeding, so long‐term use of an IABP should be avoided. Septic cardiomyopathy is a reversible condition, so an IABP may only be required for short‐term circulatory assistance until cardiac function improves spontaneously.

As a differential diagnosis, Takotsubo cardiomyopathy was not ruled out by coronary angiography. We need to accumulate additional patients with septic cardiomyopathy who receive treatment with the IABP in the future to evaluate its usefulness in more detail.

## Conclusion

Our findings suggested that the IABP could be useful for salvaging patients with septic cardiomyopathy who do not respond to conventional therapy.

## Disclosure

Conflict of Interest: None declared.
